# The Hospital Needs of Our British Forces

**Published:** 1915-05-29

**Authors:** 


### The Hospital

The Workers' Newspaper of Administrative Medicine and Institutional Life, Administration, National Insurance and Health.

No. 1510, Vol. LVIII. SATURDAY, MAY 29, 1915. PRICE ONE PENNY

### THE HOSPITAL NEEDS OF OUR BRITISH FORCES.

The present is certainly no time for carping criticism or for the multiplication of difficulties. The nation, we are told on authority, is fighting for its life, and in such circumstances we must all be prepared to make the best of circumstances. Individual interests, and even the interests of considerable classes, must, if necessary, be sacrificed for the more important end. We have on previous occasions recognised the truth of such considerations, and have admitted that they must be applied, if need be, to our civil hospitals and to our sick poor. The first claim for succour is that of our sick and wounded soldiers, and to this, if circumstances demand it, other claims must yield. Hence from the very outset we have allowed that, in the position in which the sudden outbreak of war placed us, it was right to utilise our civil hospitals for medical military purposes, even though this proceeding involved prejudice to the primary and intended purpose of these hospitals—namely, the treatment of the sick poor. We still adhere to this principle. But its application, it will be noted, is conditional on the existence of a necessity. In itself it is obviously a wrong and an injustice that institutions intended for one purpose should be used for another and different purpose and at the expense of the original beneficiaries. The evil may have to be accepted in the national interest. But it is not to be accepted with congratulation and the clapping of hands. Rather is it to be deplored and to be made a subject for inquiry with a view to determine whether it could not have been avoided. On the face of it, to take what belongs to one group of persons and to use it for others is a proceeding which needs justification, and the onus rests with those responsible for such action.

Suppose we fully admit that the necessity existed some months ago, this is very far from an acknowledgment that it exists to-day. And even if it be allowed that the conditions have not changed, there remains the question of responsibility for the stagnation. In the early days of the war we pointed out that more provision for the sick and wounded would be necessary, and we urged that this provision ought to be made, and could be made, without any serious invasion of the resources of the civil hospitals. Quite naturally at the time, managers of these institutions were abundantly ready—as every patriotic person was ready—to render all possible help. The policy, too, was pleasing to the subscribers to the hospital funds, and in some quarters it was judged good business as a means of getting new subscribers. The dispossessed sufferers were perhaps in some measure forgotten. That they were forgotten by the War Office we make no doubt; and the remark carries no reflection. Whitehall has other cares. Responsibility for the provision of hospital accommodation for the sick poor falls in another direction, and it ought not to be evaded. At the present moment it is acute, and it is likely to be more acute. The news from France carries on the face of it the certainty that very large numbers of wounded men will shortly be arriving in this country, and, indeed, the hospitals have been urged to make provision for them, and beds have been emptied accordingly. Again we repeat that if this is necessary it must be done, and if suffering is necessary it is rather the civilians than the soldiers who must endure it. But we hold strongly that it ought not to have been necessary. There has been time and opportunity and warning, and if adequate provision has not been made there is serious ground of complaint against the responsible authorities. We wish our wounded men to have the best which skill and thoughtfulness can supply, but we wish also, unless circumstances render such a course impossible, that these advantages shall equally remain for the civil population.

To write in this fashion is hardly a grateful task, and it may be an unpopular one. All the same, we feel it necessary to register a protest. Further, the necessity seems all the greater as some of the hospital authorities are inclined, we think, to take the matter too lightly. Yet upon them rests the responsibility of trusteeship, and it is theirs to see that the interests of the sick poor which have been committed to their keeping are efficiently protected.

A national emergency may be a good defence, but it is for the trustees to see that in this respect such emergency exists, and could not have been avoided. With great respect we suggest that it is the duty of hospital governors to keep before the authorities the necessity of restricting the invasion of the hospitals to the lowest possible limit, for demands readily granted, and without explanation of what they actually involve, are apt to encourage an appetite for more. The opposite course is a popular and easy one, but is it just to the other interests concerned? It is very pleasant to talk about "patriotic duty" and the wonders of our medical services in the sleek and self-congratulatory phrases which recently fell with grateful effect on the ears of the members of the Hospital Saturday Fund. But hospital officials who accept complacently the exclusion of considerable numbers of the sick poor from the benefits intended for their use seem to us to have but an inadequate conception of their duty. If such a course is necessary it ought certainly to be accepted, but it ought to be accepted with regret and not with a parade of cheap patriotism. For patriotism is cheap when it forgets the cost at which the national work is being done.

